# HPV-Induced Oropharyngeal Cancer and the Role of the E7 Oncoprotein Detection via Brush Test

**DOI:** 10.3390/cancers12092388

**Published:** 2020-08-23

**Authors:** Wegene Borena, Volker H. Schartinger, Jozsef Dudas, Julia Ingruber, Maria C. Greier, Teresa B. Steinbichler, Johannes Laimer, Heribert Stoiber, Herbert Riechelmann, Barbara Kofler

**Affiliations:** 1 Institute of Virology, Department of Hygiene, Microbiology, Social Medicine, Medical University of Innsbruck, Peter-Mayr-Strasse 4b, 6020 Innsbruck, Austria; Wegene.Borena@i-med.ac.at (W.B.); heribert.stoiber@i-med.ac.at (H.S.); 2 Department of Otorhinolaryngology, Medical University of Innsbruck, Anichstrasse 35, 6020 Innsbruck, Austria; volker.schartinger@i-med.ac.at (V.H.S.); jozsef.dudas@i-med.ac.at (J.D.); julia.ingruber@tirol-kliniken.at (J.I.); maria.greier@tirol-kliniken.at (M.C.G.); teresa.steinbichler@i-med.ac.at (T.B.S.); herbert.riechelmann@i-med.ac.at (H.R.); 3 University Hospital of Cranio-Maxillofacial and Oral Surgery, Medical University of Innsbruck, Anichstrasse 35, 6020 Innsbruck, Austria; johannes.laimer@i-med.ac.at

**Keywords:** human papillomavirus, oropharyngeal cancer, E7 oncoprotein, brush test, p16 IHC

## Abstract

*Background:* High risk human papillomavirus (hr-HPV)-associated oropharyngeal cancers (OPCs) are characterized by significantly better therapy responses. In order to implement a de-escalated treatment strategy for this tumor entity, it is highly crucial to accurately distinguish HPV-associated OPCs from non-HPV-associated ones. *Methods:* In this prospective study, 56 patients with histologically confirmed OPC were evaluated. A commercially available sandwich ELISA test system was used for the detection of hr-HPV E7 oncoprotein targeting the genotypes 16, 18 and 45. Results were presented as optical density. Positivity for HPV DNA and p16 immunohistochemistry (IHC) was taken as the reference method. *Results:* E7 positivity was significantly associated with the reference method (*p* = 0.048). The sensitivity, specificity, positive predictive value and negative predictive value for the E7 oncoptotein was 60.9% (95% CI 38.5 to 80.3%), 66.7% (95% CI 46% to 83.5%), 64.2% (95% CI 49.4 to 77.4%) and 63.01% (95% CI 48.9–75.2%), respectively, for the cutoff provided by the manufacturer. *Conclusions:* We found a significant association between E7 oncoprotein detection and the currently used combination. We believe that the use of the ELISA based E7 antigen test could be a valuable addition in cases of ambiguous findings and may be used in combination with other techniques to distinguish between HPV-driven and non-HPV-driven OPCs. However, the low sensitivity of the assay coupled with the small sample size in our study may represent a limitation. We recommend that future larger studies elucidate the diagnostic value of the E7 brush test.

## 1. Introduction

An increase in oropharyngeal cancer (OPC) was first observed in the United States at the beginning of the 21st century [1,2]. Since then, a steady increase in OPCs in the USA and Europe has been described, caused by high risk human papillomavirus (hr-HPV) [3,4], while the number of smokers and the incidence of tobacco related head and neck cancers have declined [5]. Changes in sexual behavior in the last decade, like high numbers of oral sex partners, seem to play an important etiological role in the rising incidence of HPV-positive OPCs [6]. The HPV-positive OPC is a distinct tumor entity that can be distinguished from HPV-negative OPCs by its etiology, molecular characteristics and clinical presentation [7,8]. Patients with an HPV-positive OPC have a substantially better prognosis [2,9]. In a retrospective European study of 259 OPC patients, the HPV status was the most important parameter for overall survival, regardless of the treatment strategies. HPV association was shown to positively influence the survival more than, for example, the size of the primary tumor or smoking status [7]. Hence, precise distinction between HPV-driven and non-HPV-driven tumors is essential. The detection of HPV DNA in tumor tissue does not necessarily speak of an HPV-driven tumor as only a small proportion of HPV infections lead to a transforming lesion [10]. 

This highlights the need to incorporate a diagnostic surrogate marker which precisely distinguishes between a transient and a transforming hr-HPV infection. The biomarker currently in use for the diagnosis of HPV-driven OPC is the copresence of HPV DNA and overexpression of the cellular marker p16 protein [11,12].

P16 is highly expressed in tissues undergoing cell cycle deregulation, suggesting that the detected hr-HPV DNA in the tumor tissue may be the cause of OPC. However, being merely a cellular marker, p16 is also overexpressed in lesions with no HPV association. Rasmussen and coworkers, for example, observed in a cohort of 1243 OPC patients a group of p16-positive but HPV DNA-negative patients. These patients experienced a significantly higher hazard ratio (HR) for metastatic recurrence as compared to HPV+/p16+ patients (HR = 2.56) (*p* = 0.006) [12]. This may translate into lower reliability of this marker in correctly identifying HPV-induced OPC and justifies the need to search for a surrogate marker which is closely linked to hr-HPV oncogenesis. One possible alternative is the detection of upregulated expression of hr-HPV oncoproteins. Hr-HPV oncoproteins (E6 and E7) play a major role in HPV-associated malignant transformation since they are able to inactivate tumor suppressor proteins and consequently inhibit cell cycle control mechanisms [13,14]. The molecular mechanism of the E7 oncoprotein is the inactivation of the retinoblastoma (Rb) protein—a tumor suppressor cellular protein which controls key regulators of S-phase genes. Hr-HPV E7 oncoproteins interact with Rb at a higher efficiency than low-risk HPV E7 oncoproteins. The interaction of E7 with Rb causes disruption of the growth-suppressive Rb-E2F complexes, promoting G_1_-S cell cycle transition and uncontrolled cellular replication [15,16].

A recently published study evaluated ELISA-based detection of hr-HPV E7 oncoprotein as a screening method in cervical samples of healthy women. Agorastos and coworkers found in this study that E7 oncoprotein detection might be a promising marker for precisely distinguishing transformation-relevant hr-HPV infections from transient ones [17]. The ELISA-based procedure is easy to perform, less time-consuming and requires only a basic laboratory setup. Although this sandwich-based E7 antigen ELISA test has become available commercially within the last couple of years, no previous study ever evaluated this assay among patients with OPC.

Confronted with the increasing need to establish a de-escalated therapy strategy explicitly for patients with HPV-driven OPC, we questioned whether this ELISA-based E7 oncoprotein test could be a reliable option in accurately distinguishing between HPV-driven and non-HPV-driven OPC.

## 2. Results

### 2.1. Study Population

During the study period, 56 patients with OPC were included. Of the study population, 46 (82.1%) patients were male, the mean age at diagnosis was 65.4 (standard deviation ± 10.12) years and 26 (46.4%) patients were positive for the E7 oncoprotein (Table 1). The mean follow-up time was 8.0 months ± 6 months.

In 23 (41.1%) OPC patients, HPV DNA was detected, and the most common genotype was HPV 16 (83%) (Table 2).

The E7 oncoprotein was detected in 26 patients (46.4%) and was associated with the American Society of Anesthesiologists (ASA) classification (*p* = 0.04) and smoking was inversely and significantly associated with E7 oncoprotein positivity (*p* = 0.009). Further clinico-pathological parameters are shown in Table 3.

### 2.2. *Detection of E7 Oncoprotein, HPV DNA and p16*

In 26/56 patients, E7 oncoprotein was detected; in 23/56 patients, HPV DNA was detected, and in 29/56 patients, p16 immunohistochemistry (IHC) positivity was detected. There was a high association between p16 and HPV DNA (*p* < 001) (Table 4). Patients being positive or negative for both test methods, HPV DNA and p16 IHC, served as reference method. The E7 oncoprotein was associated (*p* = 0.048) with the reference method, including 50 patients with concordant results for p16 and HPV DNA (Table 5). The two patients who were positive for HPV genotypes other than those detectable by the E7 oncoprotein ELISA tests were in one case positive (HPV 33) and in one case negative (HPV 58) for the E7 oncoprotein. 

**Figure 1:**
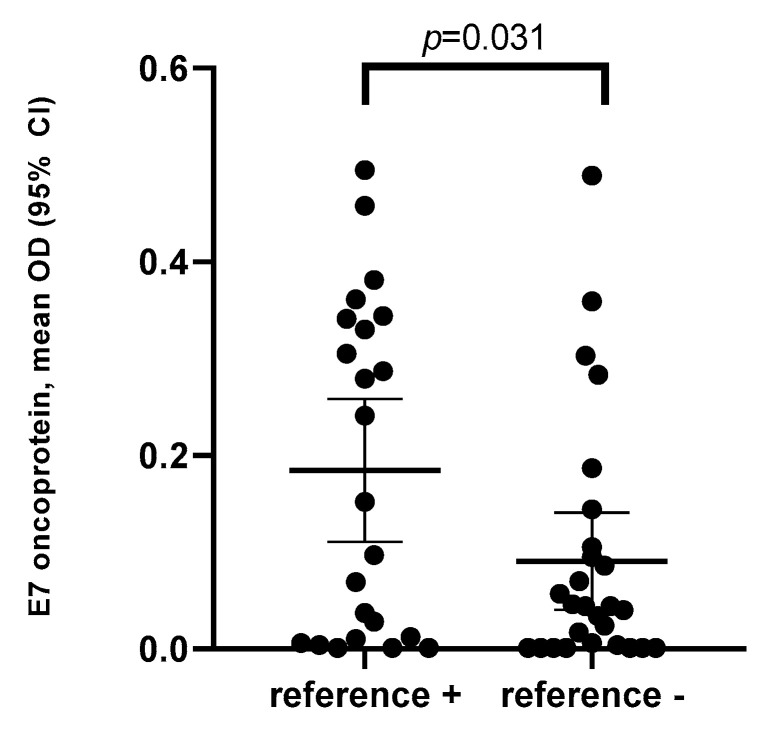
Mean optical density (OD) of the E7 oncoprotein level by the reference method (p16 and HPV DNA) among patients with histologically confirmed oropharyngeal cancer (OPC).

Mean optical density (OD) and 95% CI of the E7 oncoprotein ELISA was shown to be significantly higher among patients who were positive for the reference method (0.18, 95% CI 0.05–0.26) (median OD = 0.15) as compared to those who were negative for the reference method (0.09, 95% CI 0.05–1.13) (median OD = 0.04) (*p* = 0.031) (Figure 1). 

In addition to the cutoff value provided by the manufacturer, we also analyzed the performance of E7 oncoprotein using other arbitrarily selected cutoff points. The best agreement with the reference method is achieved when using a cutoff OD value of 0.2. With this cutoff value, the E7 oncoprotein ELISA shows the highest specificity (and the highest % agreement) as compared to the cutoff value provided by the manufacturer (Table S1).

### 2.3. *Sensitivity, Specificity and Accuracy*

**Figure 2:**
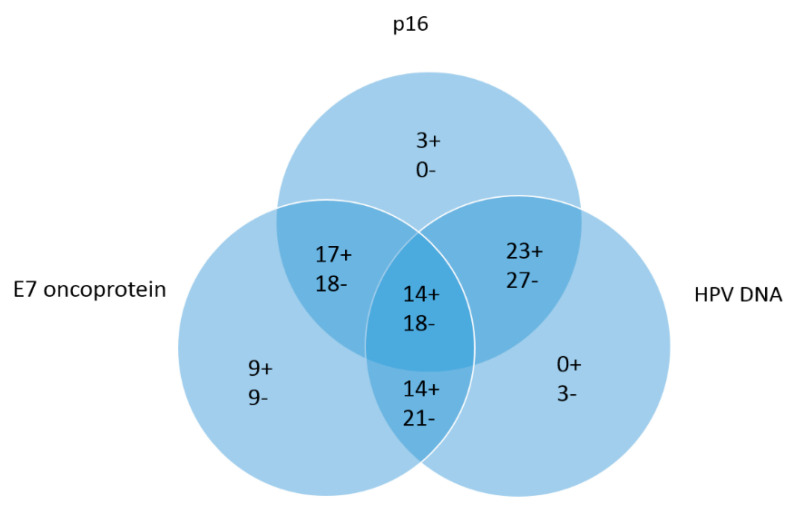
Venn diagram for E7 oncoprotein, p16 IHC and HPV DNA, (+) describing positive and (−) describing negative results. Field in the center describes concordant results for all 3 test methods (14 positive and 18 negative). The overlapping fields describe concordant results of E7 and HPV DNA (14 positive and 21 negative), E7 and p16 (17 positive and 18 negative) and HPV DNA and p16 (23 positive and 27 negative). The fields on the outside describe single positivity or negativity for the single test methods.

Sensitivity, specificity, positive predictive value and negative predictive value for the E7 oncoptotein was 60.9% (95% CI 38.5 to 80.3%), 66.7% (95% CI 46% to 83.5%), 64.2% (95% CI 49.4 to 77.4%) and 63.01% (95% CI 48.9–75.2%), respectively. The percent agreement between the standard approach and E7 method was 64%. Concordant results of the three test methods are shown in Figure 2.

## 3. Discussion

To our knowledge, this is the first study evaluating the role of E7 oncoprotein detection in patients with OPC for a precise distinction of HPV-driven from non-HPV-driven OPCs with a simple tumor surface brush sample. This test was initially developed to triage HPV-positive women being screened for cervical cancer [17]. A positive result in this assay (values greater than 0.5 pg/well (OD > 0.076)) was defined to be consistent with upregulated E7 function, corresponding to a transcriptionally relevant hr-HPV infection. Compared to p16 IHC, this ELISA-based E7 oncoprotein assay is easy to perform and less time-consuming. As different de-escalation treatment strategies to reduce toxicity in HPV-positive OPC patients are in current development [18,19,20], the detection of HPV-induced OPCs is essential. As previously mentioned, p16 is not sufficient as a single test method, particularly because it is a cell cycle deregulation marker, which may be expressed also in other tumor setups with no HPV involvement. Rb loss through a non-HPV associated mutation can result likewise in p16 expression [21]. In this case, the presence of a high level of E7 oncoprotein, the only source of which is transcriptionally active hr-HPV infection detected, may be a more specific biomarker.

Despite the low sensitivity and specificity of the assay, E7 oncoprotein detection was significantly associated with the reference method (*p* = 0.048), consisting of concordant HPV DNA and p16 results. 

E7 oncoprotein positivity was also associated with the ASA score (*p* = 0.04), indicating the lower rate of comorbidities [22] in HPV-positive patients [23]. This assumes that E7 oncoprotein detection may identify younger and healthier patients benefiting from a de-escalation therapy. Further findings were an association between the E7 oncoprotein and smoking status; the E7 oncoprotein was more often expressed in non-smokers (*p* = 0.009). This is in accordance with previous studies describing HPV-positive patients to be more commonly non-smokers, whereas smoking and alcohol consumption are the pathogenic mechanisms in non-HPV-driven OPCs [24,25,26]. There was no statistically significant difference regarding the subsite of the OPC; however, despite the low sample size of this subsite, no E7 oncoprotein expression was found in OPCs of the uvula or lateral pharyngeal wall.

Interestingly, in nine patients, a single positive result for the E7 oncoprotein was found, and also, in nine patients, a single negative result was found. The single negative ones may indicate an OPC definitely of non-HPV origin despite the presence of HPV DNA in the lesion, i.e., a non-transforming HPV co-infection. 

The single negative result of E7 could indicate episomally latent hr-HPV in the cells of a tobacco smoker and alcohol-induced carcinoma without HPV being involved in the carcinogenesis. A further explanation may be the low sensitivity of ELISA-based antigen tests, with a consequence of failing to detect low levels but yet relevant amounts of E7 oncoprotein.

The single E7-positive result may also encourage us to consider resetting the cutoff value for E7 positivity. Since the test kit was originally approved for triaging in cervical cancer screening, it may be essential to re-evaluate the assay in the context of OPC to optimize an appropriate threshold for this entity. In our study, we observed much better performance characterized by the best percent agreement and high specificity when using a higher cutoff value than provided by the manufacturer (also supported by receiver operating characteristic analysis).

Data from cervical cancer studies show that, in some tumors, although definitely caused by HPV, tests are negative for HPV DNA [27,28]. A plausible explanation is the non-productive nature of the infection in the oncogenic setup, in which viral DNA is integrated in the host. This situation, being transcriptionally active, may be characterized by high expression of transforming proteins, with less virus being released. The fact that we are taking brush samples from the surface of the tumor may support this notion. We cannot explain with certainty the single E7 positive cases. They may be due to the non-productive nature of the infection in tumors. Such phenomena are known among patients with invasive cervical cancers [29].

Moreover, in this study, a certain proportion of patients (6 patients, 10.7%) positive for p16 IHC and negative for HPV DNA was observed. The importance of additional HPV DNA testing to identify HPV-positive OPCs was described in several studies [12,30,31]. As previously described, these patients seem to have a less favorable prognosis than HPV DNA and p16-positive patients [12,32]. Weinberger and coworkers classified 79 OPC patients into class I (HPV DNA negative/p16 low positive), class II (HPV DNA positive/p16 low positive) and class III (HPV DNA positive/ p16 high positive). Class III patients had improved overall survival (*p* = 0.0095) and disease-free survival (*p* = 0.03) in comparison to class I and class II OPC patients [33]. 

Detection of the HPV oncoproteins in the clinical routine of OPC patients is still challenging. The brush test is very feasible and easily applied; however, further studies based on our results and further developments may be necessary. The brush is designed for the cervix, so, in the oropharyngeal region, a smaller brush would be more applicable in order to ensure that the tumor surface can be brushed more precisely without touching the surrounding tissue. A major limitation of the study is the lack of data on E7 mRNA expression. Taken by some as the gold standard for the classification of transcriptionally active hr-HPV, results of mRNA PCR may have supplemented the low sensitivity of this ELISA-based antigen assay. However, we used the combination of HPV DNA and p16 detection as a reference method, since this combination is the standard method for the diagnosis HPV-driven tumor according to many guidelines [11,12]. The role of routine E7 mRNA detection should, however, be elucidated in a future study. Since the great majority (>95%) of HPV driven tumors are due to HPV 16 and HPV 18 [34], the fact that our ELISA assay was limited to the three genotypes (16, 18 and 45) is a negligible limitation. A possible cross-reaction between other genotypes genetically closely linked to these three genotypes cannot be excluded. A further limitation of the study is the fact that this ELISA assay detects only E7 protein and not E6, another oncogenic protein owned by HPV which is of carcinogenic significance in a different pathway. The additional detection of E6 oncoprotein might have added valuable information to the data already generated by this study. Further studies need to elaborate on the diagnostic value of this method to additionally determine E6 oncoprotein for these hr-HPV types in order to accurately identify HPV-driven OPCs. 

## 4. Materials and Methods 

This was a prospectively designed study, and patients presenting with OPC between January 2018 and June 2020 at the Department of Otorhinolaryngology, Medical University Innsbruck, Austria were included. Each patient who agreed to participate in this study gave informed consent. The study was conducted in full accordance with the principles expressed in the Declaration of Helsinki and was approved by the ethics committee of the Medical University Innsbruck. The respective reference number was 1147/2018. 

Patients were included only when the histology confirmed squamous cell carcinoma of the oropharynx and the patient’s age was at least 18 years. Patients with other diagnoses than OPC in the histopathological examination were excluded. With the exception of 3 patients, all patients had a primary tumor in the oropharyngeal regions, and 3 patients presented with a recurrence or second primary in the oropharynx. In all patients, the E7 brush test was performed before treatment.

### 4.1. Specimen Harvest and Handling 

Details of specimen harvesting and handling has been described in a previous work [35]. In short, each patient suspected to have OPC received a panendoscopy under anesthesia. In this procedure, two different cytology brush tests for HPV DNA and E7 oncoprotein detection were conducted (digene^®^ HC2 DNA Collection Device, Qiagen, Hilden, Germany and ThinPrep^®^ PreservCyt Solution, Hologic, Manchester, UK) by gently brushing the tumor surface. The brushes were then placed in a sterile container and sent to the Institute of Virology, Medical University Innsbruck. Furthermore, tumor biopsies were obtained and fixed in formalin for routine histopathological examination at the Department of Pathology, Medical University Innsbruck. Additionally, a tumor biopsy for p16 IHC detection was kept in cell culture medium and immediately sent to the Laboratory for Molecular Biology and Oncology, Department of Otorhinolaryngology.

### 4.2. E7 Oncoprotein Detection by Brush Test 

Detection of E7 oncoprotein for genotypes of HPV 16, 18 and/or 45 was conducted using a sandwich ELISA test system (recomWell HPV 16/18/45, Mikrogen, Neurid, Germany) developed and validated (CE-labelled) initially to support diagnostic and therapeutic decisions in cervical cancer screening [17]. The ELISA microtiter plates (MTPs) were coated with rabbit monoclonal antibodies (RabMabs) specific for the three genotypes mentioned above. The remaining oropharyngeal swab samples, after cytology and HPV genotyping, were centrifuged and the pellets were incubated with the RabMabs after a couple of lysis steps. Biotinylated polyclonal goat-anti-E7 antibodies were used to detect E7 antigens which remained bound to the monoclonal antibodies after final washing steps. Results were provided as optical density (OD), with a limit of detection of 0.5 pg of protein per well, with the corresponding cutoff value of the OD being 0.076. We also evaluated the association between the reference method and E7 oncoprotein positivity using further arbitrarily selected cutoff values. 

### 4.3. DNA Amplification and HPV Genotyping 

For HPV DNA detection, real-time PCR was used based on the amplification of the L1 open reading frames (ORF). As internal control for the availability of cellular material, a PCR for the housekeeping gene beta globin was performed. The HPV DNA was considered positive if the fluorescence signal appeared before the fortieth cycle [36]. Further genotyping was performed on all HPV-positive samples using reverse line blot hybridization on nitrocellulose membrane strips containing genotype specific probes (AmpliQuality HPV-TYPE EXPRESS, AB Analitica^®^, Padova, Italy) [37]. With this genotyping kit, it is possible to identify 40 different HPV types, including all hr and several low-risk HPV types. 

### 4.4. Immunohistochemistry

First, five-micrometer thin paraffin sections were dewaxed and then antigens were retrieved in an automated staining system (Ventana, Discovery, Tucson, AZ, USA). For p16 detection, a commercial diagnostic assay was used (CINtec^®^ Histology V-Kit, Roche diagnostics, Basel, Switzerland). The staining was completed by using a universal secondary antibody solution, the DAB MAP Kit and hematoxylin counterstaining (both Ventana products). One experienced observer evaluated the tumor cell areas, and specimens were considered p16-positive if ≥66% of the cells in the tumor areas revealed immunohistochemical reaction products. 

### 4.5. Data Analysis 

Patient clinical data were presented in tabular form. A comparison of HPV DNA, E7 detection and p16 IHC in samples obtained from OPC patients was performed. For each investigated variable, a binary outcome (positive/negative) was obtained. Combined hr-HPV DNA positivity and p16 positivity served as the reference method. Contingency tables were analyzed with Fisher exact test or Pearson chi-square. Diagnostic accuracy parameters including sensitivity and specificity were calculated using the diagnostic test routines of MedCalc. For data analysis, SPSS Statistics 24 software (IBM Corporation, Armonk NY, USA) was used.

## 5. Conclusions

We do not have strong evidence that confirms that ELISA-based E7 oncoprotein replaces the reference method for diagnosis of HPV-associated OPC. However, looking at the significant association with the reference method of diagnosis, it could be a valuable addition in the case of ambiguous findings. We strongly recommend further investigation and optimization of the test in large prospective studies.

## Figures and Tables

**Table 1:** Study population.

Variables	N (%)
Male	46 (82.1%)
Female	10 (17.9%)
Mean age	65.4 years (±10.12)
E7 positivity *	26 (46.4%)

* E7 oncoprotein for human papillomavirus 16, 18 and/or 45 without differentiation.

**Table 2:** HPV genotypes in oropharyngeal cancer (OPC) patients.

HPV Subtypes	Number and Percent of HPV + OPC Patients
HPV 16	19 patients (83%)
HPV 18	2 patients (9%)
HPV 33	1 patient (4%)
HPV 58	1 patient (4%)

**Table 3:** Clinico-pathological characteristics of OPC patients.

Variables	E7 Positive (*n* = 26)	E7 Negative (*n* = 30)	*p*-Value
**Sex**			
Male	22	24	*p* = 0.73
Female	4	6	
**Age**			
≤65 years	10	10	*p* = 0.45
>65 years	16	20	
**ASA score**			
ASA I/II	19	14	*p* = 0.04
ASA III/IV	7	16	
**Smoking**			
Non-smokers	16	8	*p* = 0.009
Smoker	10	22	
**Alcohol consumption**			
Daily	8	13	*p* = 0.33
Not daily	18	17	
**Clinical T-stage**			
cT1/T2	14	16	*p* = 1.0
cT3/4	12	14	
**UICCC**			
Stage I	1	3	*p* = 0.84
Stage II	4	4	
Stage III	6	7	
Stage IV	15	16	
**Subsite oropharynx**			
Palatine tonsil	17	19	*p* = 0.14
Base of tongue	9	6	
Uvula	0	3	
Lateral pharyngeal wall	0	2	
**Therapy**			
Surgery only	3	5	*p* = 0.72
Surgery and PORT	3	4	
Surgery and RCT/RIT	1	0	
Primary RCT/RIT	14	13	
Primary RT	2	3	
Chemo only	2	3	
**p16**			
Positive	17	12	*p* = 0.05
Negative	9	18	
**HPV DNA**			
Positive	14	9	*p* = 0.06
Negative	12	21	
**Follow up**			
No recurrence	15	20	*p* = 0.55
Progression	6	9	

OPC, oropharyngeal cancer; UICC, Union for International Cancer Control; PORT, postoperative radiation; RCT, radiochemotherapy; RIT, radioimmunotherapy; RT, radiotherapy; ASA, American Society of Anesthesiologists.

**Table 4:** HPV DNA and p16.

HPV DNA	p16			
	Negative	Positive	Total	*p*-Value
Negative	27	6	33	*p* < 0.001
Positive	0	23	23	
Total	27	29	56	

**Table 5:** E7 oncoprotein and reference method.

Reference Method	E7 Oncoprotein			
	Negative	Positive	Total	*p*-Value
Negative	18	9	27	*p* = 0.048
Positive	9	14	23	
Total	27	23	50	

## Supplementary Materials

The following are available online at https://www.mdpi.com/2072-6694/12/9/2388/s1, Table S1: Comparing the performance of hr-HPV E7 oncoprotein ELISA in detecting HPV-driven OPC* (*n* = 50).
