# Supplementary material for: HPV-Induced Oropharyngeal Cancer and the Role of the E7 Oncoprotein Detection via Brush Test
Source: Cancers (Basel). 2020 Aug 23;12(9):2388. doi: 10.3390/cancers12092388 (PMC7563171; doi:10.3390/cancers12092388)
Supplement: Supplementary file 1 [file cancers-12-02388-s001.pdf]

Supplementary Materials: HPV-Induced Oropharyngeal Cancer and the Role of the E7 Oncoprotein Detection via Brush Test

Wegene Borena, Volker H. Schartinger, Jozsef Dudas, Julia Ingruber, Maria C. Greier, Teresa B. Steinbichler, Johannes Laimer, Heribert Stoiber, Herbert Riechelmann and Barbara Kofler

Table S1. Comparing the performance of hr-HPV E7 oncoprotein ELISA in detecting HPV-driven OPC* (*n* = 50).

E7 Oncoprotein OD Cutoff	Overall % Agreement (95% CI)	Sensitivity	Specificity	PPV	NPV
≥0.076**	64.0 (49.2–77.1)	60.9 (38.5–80.3)	66.7 (46.0–83.5)	60.9 (45.4–74.4)	66.7 (52.9–78.1)
≥0.1	64.0 (49.2–77.1)	52.2 (30.6–73.2)	74.1 (53.7–88.9)	63.2 (44.8–78.4)	64.5 (52.9–74.6)
≥0.15	66.0 (53.3–80.5)	52.2 (30.6–73.2)	77.8 (57.7–91.4)	66.7 (47.2–81.8)	65.6 (54.4–75.4)
≥0.2	68.0 (53.3–80.5)	47.8 (26.8–69.4)	85.2 66.3–95.8)	73.3 (50.3–88.2)	65.7 (55.7–74.5)
≥0.25	66 (51.2–78.8)	43.5 (23.2–65.5)	85.2 (66.3–95.8)	71.4 (47.5–87.4)	63.9 (54.5–72.4)
≥0.3	64.0 (49.2–77.1)	34.8 (16.4–57.3)	88.9 (70.8–97.7)	72.7 (44.3–89.9)	61.54(53.4–69.9)

HPV-driven tumor in this study is defined as the co-detection of HPV DNA and p16. ** cutoff provided by the manufacturer. OD = optical density, PPC = positive predictive value, NPV = negative predictive value.

© 2020 by the authors. Licensee MDPI, Basel, Switzerland. This article is an open access article distributed under the terms and conditions of the Creative Commons Attribution (CC BY) license (http://creativecommons.org/licenses/by/4.0/).
